# Idiopathic Adrenal Hematoma as an Unusual Cause of Acute Abdomen: A Case Report

**DOI:** 10.7759/cureus.102235

**Published:** 2026-01-24

**Authors:** Anas E Ahmed, Khalid F Alreshidi, Esraa H Al Muaddil, Shahad M Alyami, Raghad H Alsayed

**Affiliations:** 1 Community Medicine, Jazan University, Jazan, SAU; 2 College of Nursing, University of Hail, Hail, SAU; 3 College of Medicine, Najran University, Najran, SAU; 4 Faculty of Medicine, King Abdulaziz University, Jeddah, SAU

**Keywords:** acute abdomen, adrenal hemorrhage, adrenal imaging, computed tomography, conservative management, endocrine evaluation, idiopathic adrenal hematoma, nontraumatic adrenal hemorrhage

## Abstract

Idiopathic adrenal hematoma is a rare and diagnostically challenging condition that may present as an acute abdomen and closely resemble more common surgical and medical emergencies. We report a case of a 45-year-old male who presented with a sudden onset of severe abdominal pain in the absence of trauma, anticoagulant use, or any known systemic disease. Physical examination revealed localized abdominal tenderness, and routine laboratory investigations, including hematological, biochemical, coagulation, and endocrine testing, were within normal limits. Cross-sectional imaging demonstrated findings consistent with acute adrenal hemorrhage, with no evidence of an underlying functional or malignant adrenal lesion. The patient was managed conservatively with analgesia, close clinical observation, and multidisciplinary care, resulting in an uncomplicated clinical course and progressive symptom resolution. Interval imaging showed regression of the adrenal lesion, supporting the diagnosis of idiopathic adrenal hematoma. This report highlights the importance of considering adrenal hemorrhage in the differential diagnosis of acute abdominal pain, the role of imaging and hormonal evaluation in excluding secondary causes, and the utility of non-operative management with appropriate follow-up in clinically stable patients.

## Introduction

Idiopathic adrenal hematoma is a rare clinical entity characterized by spontaneous hemorrhage within the adrenal gland in the absence of trauma, anticoagulation, coagulopathy, sepsis, or an underlying adrenal neoplasm [1,2]. Adrenal hemorrhage itself is uncommon and may present as unilateral or bilateral disease, with a wide spectrum of clinical manifestations ranging from incidental imaging findings to acute abdominal pain and hemodynamic instability [2,3]. The adrenal glands are particularly vulnerable to hemorrhage due to their rich arterial supply and comparatively limited venous drainage, which predisposes them to vascular congestion and rupture under certain conditions [1-4]. However, when no precipitating factor is identifiable, the condition is classified as idiopathic, making diagnosis challenging and often a diagnosis of exclusion [2,4].

Clinically, an idiopathic adrenal hematoma can mimic more common causes of acute abdomen, leading to diagnostic uncertainty and delays in appropriate management [5,6]. Cross-sectional imaging, particularly contrast-enhanced CT, plays a crucial role in establishing a diagnosis, excluding active bleeding, and differentiating a hematoma from adrenal tumors or malignant lesions [1-4]. Equally important is the biochemical evaluation to rule out functional adrenal masses and adrenal insufficiency [5,7]. Management strategies depend on hemodynamic stability, underlying etiology, and radiologic features, with conservative treatment increasingly favored in stable patients [2-5]. Due to its rarity and nonspecific presentation, reporting such cases is essential to enhance clinical awareness, guide diagnostic evaluation, and inform optimal management approaches.

## Case presentation

A 45-year-old male patient presented to the emergency department with the sudden onset of severe right-sided abdominal pain of 12 hours in duration. The pain was described as sharp, constant, nonradiating, and progressively worsening, with a severity of 8/10 on the numeric pain scale. It was associated with nausea and a single episode of nonbilious, nonbloody vomiting. There was no history of fever, chills, bowel or urinary symptoms, trauma, strenuous physical activity, or recent abdominal procedures. The patient denied weight loss, night sweats, palpitations, episodic headaches, diaphoresis, or symptoms suggestive of catecholamine excess. There was no prior history of hypertension, bleeding disorders, anticoagulant or antiplatelet use, long-term steroid therapy, or known adrenal disease. Past medical and surgical histories were unremarkable, and there was no family history of endocrine tumors, malignancy, or coagulopathy. The patient was a non-smoker and denied alcohol or illicit drug use.

On initial examination, the patient was alert and oriented but appeared visibly distressed due to pain. Vital signs revealed normal blood pressure, a mildly elevated heart rate, and normal temperature and oxygen saturation. Cardiovascular and respiratory examinations were unremarkable. Abdominal examination demonstrated localized tenderness in the right upper and flank regions with mild guarding but no rebound tenderness or rigidity. There was no palpable mass, organomegaly, or abdominal distension. Bowel sounds were present and normal. No skin bruising or signs of trauma were observed. Examination of other systems, including the neurological and musculoskeletal systems, was within normal limits.

Initial laboratory investigations showed a hemoglobin level at the lower limit of normal without evidence of acute anemia, a normal white blood cell count, and a normal platelet count. Serum electrolytes, renal function tests, and liver function tests were within normal limits. Inflammatory markers, including C-reactive protein, were not significantly elevated. The coagulation profile, including prothrombin time, activated partial thromboplastin time, and the international normalized ratio, was normal. Given the location of the pain and the concern for possible adrenal pathology, hormonal evaluation was performed. Serum cortisol, adrenocorticotropic hormone, plasma-free metanephrines, normetanephrines, aldosterone, and renin levels were all within reference ranges, thereby effectively excluding functional adrenal neoplasms and adrenal insufficiency (Table 1).

**Table 1:** Summary of laboratory investigations at presentation and during diagnostic workup This table summarizes the hematological, biochemical, immunological, and urinary laboratory findings obtained during the patient’s initial evaluation. Reference ranges correspond to standard adult values

Laboratory test	Result	Unit	Reference range
Hemoglobin	12.8	g/dL	12.0–16.0
Hematocrit	38.5	%	36–46
Red blood cell count	4.4	×10⁶/µL	4.0–5.2
Mean corpuscular volume	87	fL	80–96
Mean corpuscular hemoglobin	29	pg	27–33
Mean corpuscular hemoglobin concentration	33	g/dL	32–36
White blood cell count	7.6	×10³/µL	4.0–11.0
Neutrophils	62	%	40–70
Lymphocytes	28	%	20–45
Monocytes	7	%	2–10
Eosinophils	2	%	1–6
Basophils	1	%	0–1
Platelet count	265	×10³/µL	150–450
Sodium	138	mmol/L	135–145
Potassium	4.2	mmol/L	3.5–5.1
Chloride	102	mmol/L	98–107
Bicarbonate	25	mmol/L	22–29
Blood urea nitrogen	14	mg/dL	7–20
Creatinine	0.9	mg/dL	0.6–1.3
Calcium	9.2	mg/dL	8.6–10.2
Aspartate aminotransferase	22	U/L	10–40
Alanine aminotransferase	25	U/L	7–56
Alkaline phosphatase	78	U/L	44–147
Total bilirubin	0.7	mg/dL	0.2–1.2
Albumin	4.1	g/dL	3.5–5.0
C-reactive protein	2.1	mg/L	<5.0
Erythrocyte sedimentation rate	10	mm/hr	<20
Prothrombin time	11.8	seconds	10–13
International normalized ratio	1.0	—	0.9–1.1
Activated partial thromboplastin time	30	seconds	25–35
Serum cortisol	16.5	µg/dL	6–23
Adrenocorticotropic hormone	32	pg/mL	10–60
Plasma free metanephrine	38	pg/mL	<65
Plasma free normetanephrine	72	pg/mL	<196
Serum aldosterone	9.5	ng/dL	4–31
Plasma renin activity	1.8	ng/mL/hr	0.6–4.3
Dehydroepiandrosterone sulfate	145	µg/dL	65–380
Random blood glucose	98	mg/dL	70–140
Lactate	1.2	mmol/L	0.5–2.2

**Figure 1:**
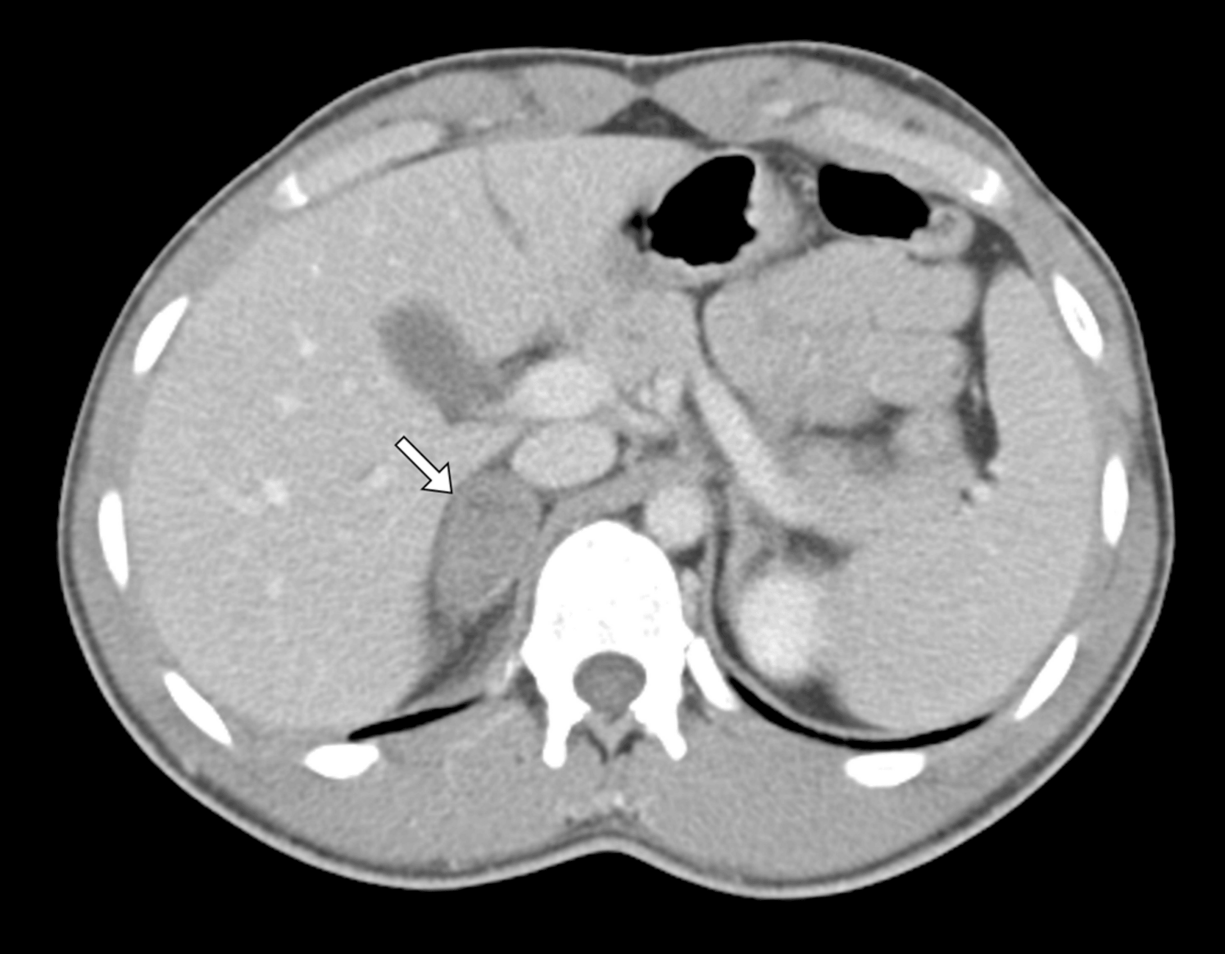
Contrast-enhanced CT demonstrating right adrenal hematoma Contrast-enhanced axial CT image of the abdomen shows a well-defined, non-enhancing lesion within the right adrenal gland (arrow). The absence of contrast enhancement and the lesion’s location are consistent with an adrenal hematoma in the appropriate clinical setting CT: computed tomography

Given the acute abdominal presentation, a contrast-enhanced CT scan of the abdomen and pelvis was performed (Figure 1). Imaging revealed a well-defined, heterogeneous, hyperdense lesion measuring several centimeters at its greatest dimension in the region of the right adrenal gland, consistent with acute adrenal hemorrhage. There was surrounding fat stranding, but no evidence of active contrast extravasation, retroperitoneal extension, or involvement of adjacent organs. The left adrenal gland was normal, and no adrenal mass, calcification, or features suggestive of malignancy were observed. There were no signs of renal stones, appendicitis, hepatobiliary pathology, or vascular abnormalities.

Based on the clinical presentation and imaging findings, the differential diagnosis included adrenal neoplasm with hemorrhage, traumatic adrenal hemorrhage, hemorrhage related to anticoagulation or coagulopathy, metastatic disease, and idiopathic adrenal hematoma. The absence of trauma, anticoagulant use, systemic disease, hormonal hypersecretion, or radiologic features of an underlying adrenal mass supported the diagnosis of idiopathic right adrenal hematoma.

The patient was managed conservatively with close monitoring. Management included adequate analgesia with intravenous followed by oral medications, antiemetics, intravenous fluids, and bed rest. Serial monitoring of vital signs and hemoglobin levels demonstrated hemodynamic stability and no evidence of ongoing bleeding. Surgical intervention or interventional radiology was not indicated, as there was no active hemorrhage or clinical deterioration. The endocrinology and surgery teams were consulted, and a consensus was reached for nonoperative management with planned radiological follow-up. During the hospital course, his abdominal pain gradually improved over several days. Oral intake was resumed without difficulty, and no new symptoms developed. Repeat laboratory investigations remained stable, with no decline in hemoglobin and no evidence of adrenal insufficiency. He was discharged home in stable condition with oral analgesics and instructions for outpatient follow-up.

**Figure 2:**
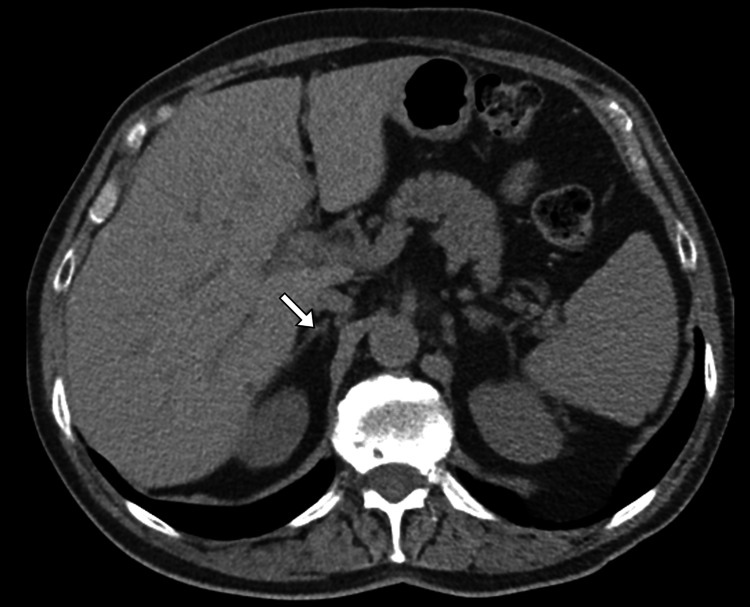
Interval resolution of right adrenal hematoma on follow-up CT Follow-up non-contrast axial image of the abdomen demonstrates near-complete resolution of the previously identified right adrenal hematoma (arrow), with no residual mass or new adrenal abnormality CT: computed tomography

A follow-up contrast-enhanced CT scan performed eight weeks later demonstrated significant interval reduction in the size of the right adrenal lesion, with resolution of hyperdensity and surrounding inflammatory changes, consistent with a resolving adrenal hematoma (Figure 2). There was no residual mass, enhancement, or features suggestive of an underlying neoplasm. At follow-up clinic visits, the patient remained asymptomatic, with complete resolution of abdominal pain and no endocrine abnormalities on repeat hormonal evaluation. The clinical, biochemical, and radiological findings confirmed the final diagnosis of idiopathic right adrenal hematoma managed successfully with conservative treatment.

## Discussion

Idiopathic adrenal hematoma is an uncommon diagnosis and remains a clinical challenge due to its rarity, nonspecific presentation, and broad differential diagnosis [2-6]. Adrenal hemorrhage has been reported in association with trauma, sepsis, anticoagulation, pregnancy, antiphospholipid syndrome, and underlying adrenal tumors. In contrast, idiopathic cases lack identifiable precipitating factors, making them diagnoses of exclusion [5,6]. This case highlights the importance of maintaining a high index of suspicion for adrenal pathology in patients presenting with acute abdominal or flank pain, even in the absence of classic risk factors.

The pathophysiology of spontaneous adrenal hemorrhage is not completely understood [5-9]. The adrenal glands possess a unique vascular anatomy characterized by multiple arterial feeders converging into a single central vein, which predisposes them to venous congestion and hemorrhage during periods of increased adrenal blood flow or venous outflow obstruction [5,7]. Proposed mechanisms in idiopathic cases include transient adrenal vein thrombosis, sudden increases in intra-abdominal pressure, stress-induced catecholamine surges causing vasoconstriction and subsequent reperfusion injury, or unrecognized microvascular fragility [2,8].

Clinical presentation varies widely, ranging from asymptomatic incidental findings to acute abdomen and, rarely, hemorrhagic shock [4,6,8]. Unilateral adrenal hematomas typically present with localized abdominal or flank pain, whereas bilateral involvement raises concern for acute adrenal insufficiency [7-12]. The absence of hypotension, electrolyte disturbances, or biochemical evidence of adrenal insufficiency in this patient supported a favorable prognosis and allowed for conservative management, underscoring the necessity of early endocrine assessment in all suspected cases [3,5,7].

Imaging plays a central role in diagnosis, management, and follow-up [4-8]. Contrast-enhanced CT is the first-line modality in the acute setting due to its wide availability and ability to detect hemorrhage, assess lesion density, and identify active extravasation [6,8]. Acute adrenal hematomas typically appear as hyperdense, non-enhancing lesions, with density decreasing over time as the hematoma resolves [3,5]. Differentiating hematoma from hemorrhagic adrenal neoplasm is critical. Magnetic resonance imaging is valuable in equivocal cases, particularly for tissue characterization and detection of underlying masses once acute blood products resolve [3,7]. In this case, interval resolution on follow-up CT without residual enhancement or mass effect strongly supported the diagnosis of idiopathic adrenal hematoma.

Management strategies depend on hemodynamic stability, laterality, and suspicion of underlying pathology [1-5]. Hemodynamically stable patients without evidence of ongoing bleeding or malignancy are best managed conservatively [3,6]. Surgical intervention or transarterial embolization is reserved for patients with active hemorrhage, hemodynamic instability, enlarging hematomas, or confirmed adrenal neoplasms [4,6,8]. Premature adrenalectomy in the acute phase should be avoided when malignancy is unlikely [1,5].

Long-term follow-up is essential to ensure complete resolution and exclude an occult adrenal tumor masked by hemorrhage [4,5]. Follow-up imaging at 6-12 weeks is recommended, with continued surveillance if abnormalities persist [4,6]. Complete radiologic resolution, as demonstrated in this patient, effectively rules out underlying neoplasia and confirms the idiopathic nature of the hemorrhage.

## Conclusions

Idiopathic adrenal hematoma is an uncommon but clinically important cause of acute abdominal pain that requires a high index of suspicion and a systematic diagnostic approach. Imaging and hormonal evaluation play a central role in confirming the diagnosis and excluding secondary causes. In clinically stable patients, conservative management with close clinical observation and follow-up imaging is both safe and effective. As a diagnosis of exclusion, idiopathic adrenal hematoma requires careful follow-up to confirm resolution and to exclude underlying adrenal pathology, thereby ensuring optimal patient care.
